# Cross-cultural adaptation, reliability, and validity of the Vertigo symptom scale–short form in the central Kurdish dialect

**DOI:** 10.1186/s12955-019-1168-z

**Published:** 2019-07-17

**Authors:** Sherko Saeed F. Zmnako, Yousif Ibrahim Chalabi

**Affiliations:** Department of Surgery-Otolaryngology, College of Medicine, University of Sulaimani, Presidency 1 Tasluja Street 501, P.O. Box: 334, Sulaimani, Kurdistan Region, Iraq

**Keywords:** Vestibular disorders, Psychometric properties, Non-normal distribution, Factor analysis, Polychoric correlation, Partial least squares, Vertigo and dizziness, Patient−reported outcome measures

## Abstract

**Background:**

Core vestibular symptoms are vague, hard for patients to describe, and difficult for examiners to quantify. Reliable and validated patient-reported outcome measures (PROMs) have obtained acceptance and popularity in the specialty of vestibular disorders. In Kurdish, there is a critical shortage of such measures. The aim of this survey was to assess the psychometric properties of a central Kurdish version (VSS − SF − CK) of the Vertigo Symptom Scale−Short Form (VSS − SF).

**Methods:**

The study utilized a regulated process of cross-cultural adaptation to produce the VSS − SF − CK. We examined its psychometric properties by using a cross-sectional survey. Owing to a non-normal distribution, both principal axis factoring and polychoric correlation were used to examine the structure. The internal consistency of the scales was evaluated using Cronbach’s alpha coefficient (α) and composite reliability. The discriminant validity was evaluated using the heterotrait–monotrait ratio of correlations (HTMT_.85_) and the Fornell-Larcker criterion. To assess convergent validity, the instrument was correlated with two comparators.

**Results:**

The participants (*n* = 195) were composed of 165 patients with vestibular symptoms (mean − age 45 ± 15.8, range 61 years; 56.4% women) and 30 healthy participants (mean − age 35 ± 18.6; range 52 years; 60% women). Based on the scree plot, along with other criteria such as Horn’s parallel analysis and minimum average partial, two factors were extracted: vestibular (VSS − V) and autonomic-anxiety (VSS − AA). Both constructs showed a robust structure in terms of adequate loadings and weak cross-loadings. The scales’ αs were 0.81, 0.81, and 0.87 for VSS-V, VSS-AA, and the total scale (VSS − T), respectively. Discriminant validity was established with a value of 0.71 for HTMT (< 0.85). Spearman’s correlation supported the study’s hypotheses and confirmed the convergent validity. Intraclass correlation coefficients revealed high external reliability: test-retest results were 0.93, 0.94, and 0.97 for VSS-V, VSS − AA, and VSS − T, respectively.

**Conclusion:**

Given a critical shortage in PROMs for the vestibular field, the psychometric properties of VSS − SF − CK were evaluated. The results were promising, as they revealed external consistency and construct validity. The goodness of fit indices showed that the VSS − SF − CK is a reliable and validated PROM that can be used by clinicians and researchers in the Kurdish-speaking population.

**Electronic supplementary material:**

The online version of this article (10.1186/s12955-019-1168-z) contains supplementary material, which is available to authorized users.

## Background

Vestibular disorders (VD) produce a group of vestibular symptoms (VS) as well as a range of concomitant autonomic-anxiety symptoms [1]. Epidemiological data on VD in the general population are scarce. Studies have reported a discrepant range (6.1 to 27%) for one-year prevalence of VS [2]. However, they are prevalent among individuals visiting outpatient care centers [3]. VS are vague and present themselves in different patterns (acute, episodic, and chronic) [4]. That is, they are difficult for patients to describe, and hard for healthcare professionals to evaluate [5]; hence, they place a burden on both patients and community [6].

One potential way to overcome the difficulty of evaluating demanding symptoms is the utilization of patient-reported outcome measures (PROMs) through reliable and validated questionnaires, which has gained acceptance and popularity in different fields of medicine [7]. Based on the Consensus-based Standards for the Selection of Health Status Measurement Instruments (COSMIN) checklist of property measurements [8], the clinical utility of a group of PROMs related to VD was appraised through a systematic review; among them, the long form of the Vertigo-Symptom Scale earned the second highest score [9]. It was developed by Yardley et al. [10] and contains 34 items. However, Mendel et al. [11] found that utilizing the long form as a single aggregated scale may result in methodological bias; to overcome this hazard he suggested studying these items separately by using the short form (VSS **−** SF).

The VSS **−** SF is composed of 15 items [12], extracted from the long form. This self-rated questionnaire uses five-point scales ranging from 0 to 4, with response options of never, a few times, several times, quite often, and very often. The score indicates the frequency of the 15 symptoms, which range from 0, suggesting no symptoms, to 60, representing persistent symptoms. According to the types of symptoms, the 15 items are divided into two subscales: vestibular (balance) (VSS **−** V), and autonomic-anxiety (VSS − AA) [13].

However, to use a PROM in a population with a language different from the source, it must undergo a process of cross**−**cultural adaptation, which includes both translation and cultural adaptation. However, translation of any validated PROM can debilitate its psychometric properties; therefore, consistency and validity should also be confirmed and reported in accordance with international guidelines for measuring patient-reported health outcomes [14]. The psychometric properties of the VSS − SF were assessed when Norwegian and Japanese versions were cross-culturally validated; both translated versions had acceptable internal consistency, external reliability, convergent validity, and discriminating validity. Two factors were explored in the Norwegian version: VSS-V and VSS-AA [15]; however, a third factor related to duration of symptoms was also extracted from the Japanese version [16].

Unfortunately, there is a critical shortage of validated tools in Kurdish that can quantify vestibular disorders. The VSS-SF is efficient, simple, short, and has not been adapted to Kurdish. Accordingly, in this study we applied an adjusted translation and cultural adaptation of the VSS **−** SF to the central Kurdish dialect (VSS **−** SF **−**CK). Utilizing a cross-sectional survey, and in accordance with the COSMIN checklist [8], we assessed the psychometric properties of the VSS − SF − CK.

## Methods

### Cross-cultural adaptation (CCA)

#### The focus group (FG)

In accordance with international regulations for qualified PROMs [8], the College of Medicine – University of Sulaimani (hereafter, “the institute”) assembled a FG, consisting of seven otolaryngologists (including one of the authors) who were all native speakers of the target language with 15 to 25 years of experience in the field of VD. The moderator of the group was aware of how to run the discussion sessions according to the corresponding guidelines [17].

#### Preparation:

Preparation consisted of three steps.

1. The corresponding author contacted and confirmed the permission of Professor Lucy Yardley as one of the original developers.
2. A junior otolaryngologist (who could easily contact the members of the FG and the translators) was recruited to follow the translation process.
3. The concepts of clarity, fluency, and unambiguity in the forwarded translations were agreed upon and followed during CCA.

#### CCA:

The process was conducted according to the steps recommended by Wild and colleagues [18] and Beaton and colleagues [19]. Two forward-translations of the contents were performed by an expert native otolaryngologist (T1) and a licensed native translator (T2). The FG compared and resolved differences between T1 and T2; then, a preliminary form of VSS **−** SF **−** CK was created (T12). After back-translation, identified discrepancies (see Additional file 1) were resolved (e.g., a clause was added to clarify the meaning of “dizziness.”) To examine the clarity, we conducted a pilot test with 18 linguistically−knowledgeable patients with vestibular symptoms. Utilizing a specific form designed for ratings (Additional file 2), members of the FG and participants in the pilot test were asked to give feedback on understandability and to rate the contents of each translated item. The CCA process and results of the ratings were reviewed; consequently, the face and content validity were considered excellent. Ultimately, after proofreading and cognitive debriefing, the final version was established (Additional file 3) and the details of the process were reported to the institute.

### Sample size and participants

Based on a subject-to-variable ratio of a minimum of 10 participants for each item [20] and factors extracted in previous research on the same instrument [16], we estimated that 165 participants would be sufficient to observe the covariation among our 15 surface attributes; along with 30 healthy control participants for comparison. Two well-equipped audio-vestibular tertiary clinics that cover a major proportion of the center and districts of Sulaimani-Governorate, Iraq enrolled participants from March 2017 to July 2018. Participants were patients with chief complaints of VS who had been objectively diagnosed as having VD.

Inclusion criteria allowed native speakers with sufficient communication and performance abilities. The exclusion criteria were: age below 17 or above 79, symptoms of less than 1 day duration (Patients needed to have experienced symptoms [a feeling of being dizzy, disoriented, or swimmy lasting all day] for at least 1 day in order to answer item-6), musculo-skeletal diseases and symptoms primarily due to other systems disorders such as neurological, cardiopulmonary, and cognitive disorders.

#### Subgroups:

The heterogeneity of symptoms in the instrument required patients with different presentations and from different settings [10]; consequently, the inclusion and exclusion criteria were adjusted to ensure that the sample was a good representation of the target population (patients with VS of vestibular origin with no associated illnesses that may produce VS). The sample contained all types of patients that may be encountered in primary, secondary, and tertiary clinics. Furthermore, based on the patterns of presentation, and to evaluate the discriminating validity, the sample was classified into three subgroups: (1) Acute presentation (acute episode of symptoms at the time of rating), (2) Chronic presentation (long-term sensations of symptoms), and (3) Episodic presentation (recurrent symptoms with symptom-free intervals) [21]. For the 76 participants who were randomly selected from the patients included in the reliability subgroup, the design was converted to a short-term longitudinal study to assess external reliability.

### Educational level and raters

The VSS − SF − CK is a self-rated survey tool, that is, the role of the rater (interviewer) is trivial [22], but not everyone in the target population is literate, so participants’ educational levels were documented. Methodologists also recommend the involvement of a female interviewer to simplify the process, considering participants’ psychological and/or societal obstacles [23]; that is, female interviewers can interview both genders, particularly women in conservative or religious families. Hence, two female raters with similar qualifications and sufficient training were recruited.

### Recruitment and randomization

While patients were waiting for the results of their investigations or rehabilitation protocols, a systematic numbered sample was used on a daily basis to select patient participants who fulfilled the inclusion criteria and accepted the invitation. The first participant was selected randomly followed by fixed-interval selection.

### Comparators

To the best of our knowledge, there are no validated PROMs in Kurdish that measure the construct under investigation. Consequently we employed two comparators that could measure a similar construct but using two different approaches, that is, subjective and objective. First, in the subjective approach, a visual analogue scale (VAS) was applied so patients could rate their total self-perceived vestibular symptoms (VAS − T). The scale started with zero to represent no symptoms and ended with 100 to represent subjectively rated worst-possible symptoms. Second, in the objective approach, the Tandem Romberg (TR) was utilized, participants were requested to maintain balance for 60 s under the following four conditions: 1- right foot behind the left, eyes open; 2- same as the first, eyes closed; 3- left foot behind the right, eyes open; 4- same as the third, eyes closed. Only one of three trials was administered for each condition if the patient could complete 60 s successfully. The scores from all four conditions (TR − T) were summed out of 240 s [24].

### External reliability

Steps recommended by Kottner and his colleagues were followed during reliability assessments and reporting [25]. Patients in the reliability subgroup (*n* = 76) were rated on two separate occasions. The timing of the second rating was arranged according to the patients’ availability.

The following strategies were used to minimize measurement errors:

1. Participants with unstable conditions (dramatic recovery or deterioration) were excluded from the reliability tests.
2. The time interval between ratings was one to 5 days; furthermore, to avoid recall bias, the sequence of items for the second rating was different. However, the interval for Tandem Romberg was one to 2 hours to remove the effect of in-between rehabilitation.
3. Similar settings were applied to all patients; ratings were performed in a quiet room to eliminate distractions and minimize auditory stimuli, so patients could not maintain their balance using these stimuli, especially in eye closed conditions (to test vestibular system alone, the role of other systems, that could help in maintaining balance, should be excluded).
4. Raters were instructed not to prompt patients for specific answers.

### Statistics

#### Data screening

Ceiling and floor effects were absent, while the percentages of patients with the highest and lowest scores in the three outcome measures were below 15% [26]; pairwise exclusion was used with missing values. In our sample size (50 < *N* < 300), absolute Z-scores above 3.29 were considered to reflect a non-normal distribution [27]. Univariate and multivariate (Mardia test) statistics revealed an asymmetric distribution. Ordinal variables such as Likert-type items fail to assume normality [16, 28] and therefore require either log-transformation or distribution-free (e.g., nonparametric) tests; we chose the latter [29].

#### Structural validity

##### Exploratory factor analysis (EFA)

To identify the latent constructs, considering a sample size of (≤300) and non-normality [20, 28], the authors conducted EFA. Some methodologists recommend use of parametric tests even if the distribution is non-normal [30]. However, for ordinal data and non-normality, others advocate more robust tests, such as polychoric correlations (PC) [31], specifically, Robust Diagonally Weighted Least Squares (DWLS) [32]. In view of the study context, principal axis factoring (PAF) was considered to outweigh maximum likelihood [28]. To certify that the same outcomes would be reproduced, and in light of the above circumstances, we utilized both PAF and DWLS in EFA. Assuming moderate inter-factor correlation (IFC), promax oblique rotation (Kappa = 4) was employed.

##### Number of factors to retain

To avert bias, guidelines emphasize using diverse strategies for finding the ultimate number of internal attributes [28, 33]. This was resolved based on five parameters:

1. Kaiser Criterion (eigenvalue > 1).
2. Scree plot.
3. Horn’s parallel analysis (HPA) [34].
4. Minimum average partial (MAP).
5. The a priori hypotheses that the instrument consists of two subscales: VSS − V and VSS − AA [15, 16].

##### Discriminant validity (internal discrimination)

To establish this feature, four criteria were utilized:

1. Cross-Loadings Inspection: Item−loading on its construct should be higher than its cross-loadings.
2. Fornell-Larcker: The average variance extracted (AVE) by each factor should be higher than the square of IFC (IFC^2^).
3. The heterotrait-monotrait ratio of correlations (HTMT) Value < 0.85 is favorable.
4. HTMT−Inference: value < 1 is assuring [35].
  The last two variables were estimated by the partial least squares (PLS) [36].

##### Model fit

This was appraised by a comparative fit index (CFI) value of ≥0.95 and the root mean square error of approximation (RMSEA) value of ≤0.06 [37].

#### External reliability

Intraclass correlation coefficient (ICC) was utilized. Cut-off values for strength of reliability were: < 0.5─poor, from ≥0.5 to ≤0.75─moderate, from ≥0.75 to ≤0.9─good, and > 0.9─excellent [22].

#### Internal consistency reliability

The following seven variables were estimated and compared with the corresponding cut-off points:

1. Cronbach’s alpha (**α**): > 0.7 [38, 39].
2. Average Inter-item correlation (AIC): ≥0.2 ≤ 0.5 [26].
3. Corrected Item-total correlation (CI − TC): ≥0.4
4. Alpha if item deleted (AIID): the resultant **α** of the selected scale should not rise if any item is deleted [38].

Methodologists consider **α** to be a controversial estimate; accordingly, the following was also reported:

(5) The consistent reliability measure of the partial least squares (rhoA): > 0.7.
(6) Composite reliability (rhoC): > 0.7.
(7) AVE by each factor: > 0.5 [40].

#### Hypotheses

Yardley stated that PROMs are cumulative measures, while objective tests are single-point measures [10]. Thus, we may find adequate correlations between subjective scores if they measure the same construct; however, the concept is not the same when subjective and objective scores are correlated even if they are measuring similar constructs [15, 41, 42]; accordingly, the following hypotheses were formed:

- The positive correlation between the total score of VSS **−** SF **− ** CK (VSS **−** T) and the VAS − T would be adequate, because they measure similar constructs with similar approaches.
- The correlation between TR-T and VSS-V scores would be moderate because they measure similar constructs with different approaches; furthermore, the value would be negative (moderately negative) because low scores on TR-T are associated with high scores on VSS-V.
- The negative correlation between TR **−** T and the VSS **−** AA would be weak because they measure different constructs with different approaches. Rank coefficient (Spearman) was used to estimate the correlations. The study classified values from assorted regulations as follows: < 0.3─weak, ≥0.3 < 0.5─moderate, ≥0.5 < 0.7─adequate, and ≥ 0.7─high correlations [16, 43].

#### Discriminating validity (external discrimination)

It is assumed that the instrument has the ability to discriminate between subgroups as well as between the patient and healthy groups. The Mann-Whitney U test was used to test this assumption with a significance level of 5%.

**Fig. 1:**
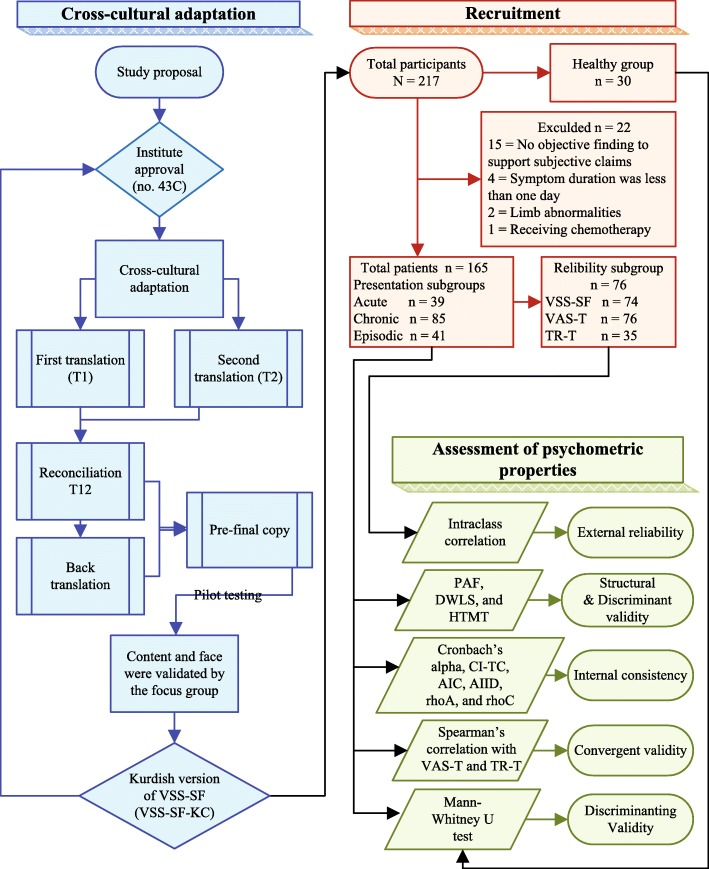
The course of the study. Note: Each color represent a specific field of work in the study; Black arrows show the sequential order and connections between the fields. Abbreviations: VSS − SF/CK, Vertigo Symptom Scale−Short Form/Central Kurdish; VAS − T, Visual Analogue Scale−Total; TR − T, Tandem Romberg−Total; PAF, Principal Axis Factoring; DWLS, Diagonally Weighted Least Squares; HTMT, Heterotrait-monotrait ratio; CI − TC, Corrected Item-Total Correlation; AIC, Average Inter-item Correlation; AIID, Alpha If Item Deleted; rhoA, Reliability measure of the partial least squares; rhoC, Composite reliability

The flowchart (Fig. 1) illustrates the sequential order of the works implemented in the study.

More details on methodology are available in Additional file 1.

### Software

Three programs were utilized: 1- FACTOR V10.8.04 (Rovira i Virgili University, Tarragona, SPAIN) for PC, HPA, and goodness of fit [44]; 2- SmartPLS 3. (Boenningstedt: SmartPLS GmbH) [36] for rhoA and discriminant validity; and 3- IBM SPSS Statistics V21 (IBM, Armonk, NY, USA) for the rest of the analysis such as, PAF, α and syntaxes for HPA and MAP [45].

## Results

Data related to participants and exclusions are presented in Fig. 1; no valid differences in the results were exhibited based on exclusions. Furthermore, more details of participants’ attributes are shown in Table 1.

**Table 1:** Demographic attributes of the groups and subgroups

	Total Patients		Reliability subgroup		Presentation subgroups^a^						Healthy group	
					Acute		Chronic		Episodic			
	*n* = 165		n = 76		*n* = 39		*n* = 85		*n* = 41		*n* = 30	
	n	%	n	%	n	%	n	%	n	%	n	%
Women	93	56.4	38	50	21	53.8	53	62.4	19	46.3	18	60
Age (year)^b^	45	±16	45	±17	45	±15	42	±16	53	±13	35	±18.6
Duration^bc^	4.5	±11.8	4.1	±14.7	0.5	±0.13	7.1	±14.9	3	±8.6		
Educational Level												
No or Primary^d^	92	55.8	43	56.6	21	53.9	41	48.3	30	73.2	5	16.7
Secondary^d^	42	25.5	19	25.0	9	23.1	28	32.9	5	12.2	20	66.7
Graduate & Post graduate	31	18.8	14	18.5	9	23.1	16	18.9	6	14.6	5	16.6
Diagnosis												
Labyrinthitis	1	0.5	1	1.3	1	2.6	0	0	0	0		
BPPV	17	8.7	7	9.2	2	5.1	0	0	15	36.6		
MD	18	9.2	11	14.5	2	5.1	4	4.7	12	29.3		
UPVH	59	30.2	28	36.8	32	82	18	21.2	9	22		
VM	15	7.7	5	6.6	2	5.1	9	10.6	4	9.8		
Other VD^e^	55	28.2	24	31.6	0	0	54	63.5	1	2.4		

**Note:** ^a^Nature of the symptoms at the time of rating not related to disorders or syndromes; ^b^Mean and ± Standard Deviation; ^c^Duration in month; ^d^Schools; ^e^No specific diagnosis could be identified

**Abbreviations:** *BPPV* Benign Paroxysmal Positional Vertigo, *MD* Meniere’s Disease; *UPVH* Unilateral Peripheral Vestibular Hypofunction, *VM* Vestibular Migraine, *VD* Vestibular Disorders

**Fig. 2:**
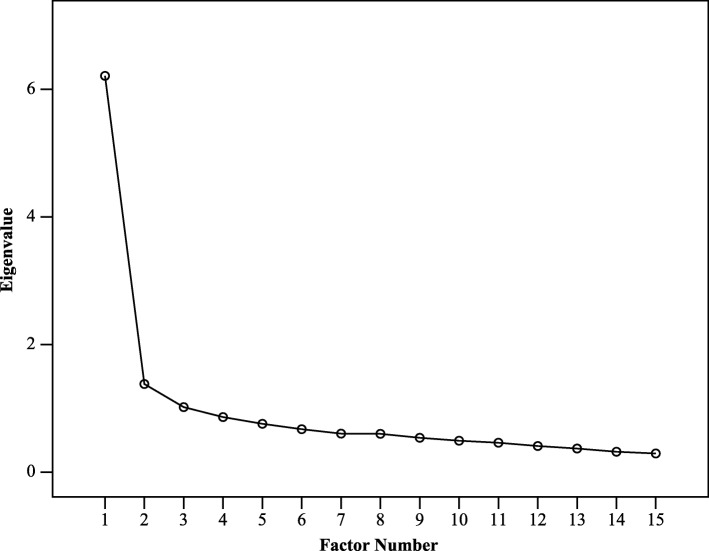
Scree plot of the initial exploratory factor analysis, based on Eigenvalues > 1. Note: The flexion of the elbow at the second factor is maximal denoting 2 factors retaining

Factorability was achieved, the determinant was not equal to zero (0.007), the Kaiser-Meyer**─**Olkin test was meritorious (0.873), and Bartlett’s test of sphericity was significant (*p* < 0.001). Based on eigenvalues > 1, PAF revealed three factors. On this basis, a 3-factor solution was applied using DWLS. The cumulative proportions of variance (CPV) in the three factors were 53 and 59% in PAF and DWLS, respectively. In the case of DWLS, the three consecutive eigenvalues and the CPV were 6.2 (41%), 1.6 (52%), and 1.1 (59%). Nonetheless, the elbow of the scree plot was distinctly flexed at the point where the second factor was located (Fig. 2). Furthermore, HPA, MAP “See Additional file 4: Table S1 and Table S2”, and the a priori hypothesis also supported the scree plot display; that is, a 2**–**factor solution.

Consequently, a 2**–**factor solution was conducted with both PAF and DWLS. Two factors were extracted: vestibular (VSS **−** V) and autonomic-anxiety (VSS **−** AA), In the case of DWLS, the two consecutive eigenvalues and the CPV were 6.1(41%), 1.6 (52%). Each factor adequately loaded seven items with weak cross-loadings. The remaining Item**–**12 (feeling faint, about to black out), was loaded adequately by the VSS **−** AA; however, it was associated with noticeable cross loadings by VSS **−** V.

The AVE by neither method reached the acceptable level, as it was < 0.5 for both factors. Additional file 5 shows how to estimate AVE and rhoC. To assess the negative effects of low AVE on discriminant validity, AVE and IFC^2^ were compared (Fornell-Larcker criterion). In PAF, the AVE by both factors were lower than IFC^2^ (validity not established); while for DWLS, AVE was higher than IFC^2^ only in VSS **−** V (validity of one factor established). However, the validity was confirmed by HTMT value = 0.71 (< 0.85) and HTMT-inference value = 0.81 (< 1). To examine the situation, we deleted item**−** 12 (the cross-loading item); consequently, in DWLS, the AVE by VSS–AA was slightly inflated and became more than a slightly deflated IFC^2^; hence, the Fornell-Larcker criterion was also achieved for the VSS **−** AA (Table 2). Additional file 6 shows the details of 2-factor extraction by DWLS and the results of model fit, CFI = 0.985 (≥0.95) and RMSEA = 0.049 (≤0.06).

**Table 2:** Item loadings in exploratory factor analysis with 2–factor solution and the internal consistency variables

	Kurdish Sample^a^								Norwegian Sample^b^	
	n = 165								*n* = 509	
	Internal consistency variables				Polychoric Correlations^c^		Principal Axis Factoring^d^		Maximum Likelihood^e^	
	CI-TC in subscales	AIID in subscales	CI-TC in total scale	AIID in total scale	Factor 1 Vestibular	Factor 2 Anxiety	Factor 1 Vestibular	Factor 2 Anxiety	Factor 1 Vestibular	Factor 2 Anxiety
**VSS–V**		**0.809**								
4- Vertigo (> 20 min)	0.56	0.783	0.49	0.862	0.91	−0.17	0.76	−0.15	0.84	− 0.18
10- Unsteady (> 20 min)	0.63	0.768	0.58	0.857	0.85	−0.06	0.76	−0.05	0.80	−0.01
13- Unsteady (< 20 min)	0.60	0.773	0.56	0.858	0.74	− 0.03	0.72	−0.04	0.58	0.14
6- Dizziness (all day)	0.59	0.777	0.61	0.855	0.58	0.21	0.53	0.19	0.81	−0.10
8- Difficult to stand or walk	0.45	0.800	0.41	0.865	0.54	−0.03	0.52	−0.03	0.67	0.07
15- Dizziness (< 20 min)	0.55	0.784	0.55	0.858	0.54	0.18	0.47	0.19	0.60	0.10
1- Vertigo (< 20 min)	0.44	0.801	0.43	0.864	0.52	0.04	0.46	0.05	0.61	0.09
**VSS–AA**		**0.807**								
9- Difficulty in breathing	0.57	0.779	0.52	0.860	−0.05	0.78	−0.07	0.69	0.02	0.55
14- Chest pain	0.46	0.794	0.40	0.865	−0.10	0.71	−0.14	0.63	0.05	0.45
7-Headache	0.51	0.787	0.46	0.863	−0.09	0.69	−0.11	0.66	0.33	0.33
11- Excessive sweating	0.55	0.781	0.53	0.860	0.06	0.59	0.06	0.56	0.09	0.82
3- Nausea, vomiting	0.52	0.785	0.50	0.861	0.05	0.59	0.07	0.52	0.35	0.31
2- spells of cold or hot	0.49	0.790	0.51	0.861	0.07	0.56	0.12	0.47	−0.02	0.81
5- Heart fluttering	0.51	0.788	0.54	0.859	0.20	0.50	0.16	0.48	−0.04	0.56
12- Feeling faint	0.55	0.781	0.62	0.855	0.33	0.45	0.30	0.43	0.43	0.32
**VSS–T**				**0.868**						
AVE					0.47	0.38	0.38	0.32		
IFC (IFC^2^)					0.63	(0.40)	0.65	(0.42)	0.56	(0.31)
RhoC					0.86	0.83	0.80	0.78		
RhoA^f^					0.82	0.82				
**If item**–**12 deleted**^**g**^										
AVE					0.47	0.40	0.37	0.33		
IFC (IFC^2^)					0.62	(0.38)	0.62	(0.39)		
RhoC					0.85	0.82	0.80	0.77		
AIC	VSS–V = 0.38			VSS–AA = 0.34			VSS–T = 0.31			

**Note:** For convenience, symptoms shortened; Alphas of the subscales and total scale are in bold and in three decimal places, to be compared with resultant alpha when any item deleted; ^a^Promax, Kappa = 4; ^b^Wilhelmsen K, Strand LI, Nordahl SHG, Eide GE, Ljunggren AE. Psychometric properties of the Vertigo symptom scale - Short form. BMC Ear Nose Throat Disord. 2008;8(1):2; ^c^Polychoric algorithm by Diagonally Weighted Least Squares (DWLS); ^d^Promax with Kaiser normalization in 3 iterations; ^e^Oblimin, Delta = 0; ^f^Values provided by SmartPLS 3; ^g^Inflation of AVE and deflation of IFC^2^

**Abbreviations:** *VSS–V/AA/T* Vertigo Symptom Scale–Vestibular/Autonomic-Anxiety/Total, *CI-TC* Corrected Item-Total Correlation, *AIID* Alpha If Item Deleted, *AVE* Average Variance Extracted, *IFC* Inter-Factor Correlation, *IFC*^*2*^ Square of IFC, *RhoC* Composite reliability, *RhoA* Reliability measure of the partial least squares, α Cronbach’s alpha, *PLS* Partial Least Squares, *AIC* Average Inter-item Correlation

Moreover, Table 2 presents the outcomes for the internal consistency variables, they were satisfactory for all methods and scales; regarding AIID, resultant α did not increase when any item was deleted. In both methods, values of rhoA and rhoC gained the acceptable limits.

The instrument and the comparators exhibited good to excellent reliabilities in all types (Table 3).

**Table 3:** External reliability of the instruments

	VSS − SF − CK *n* = 74						*n* = 76		*n* = 35	
	VSS − V		VSS − AA		VSS − T		VAS − T		TR − T	
	ICC^a^	n	ICC^a^	n	ICC^a^	n	ICC^a^	n	ICC^a^	n
Intra-rater1	0.88	28	0.93	28	0.95	28	0.98	28	0.95	12
Intra-rater2	0.83	28	0.96	28	0.97	28	0.90	29	0.80	13
Inter-rater	0.97	18	0.93	18	0.97	18	0.96	19	0.91	10
Test-retest	0.93	74	0.94	74	0.97	74	0.96	76	0.90	35

**Note:** ^a^Intraclass correlation coefficient: the model, two-way mixed effects; the type, mean of k raters; and the definition, absolute agreement

**Abbreviations:** *VSS–SF–CK/V/AA/T* Vertigo Symptom Scale–Short Form–Central Kurdish/Vestibular/Autonomic-Anxiety/Total, *VAS–T* Visual Analogue Scale–Total, *TR–T* Tandem Romberg–Total

**Fig. 3:**
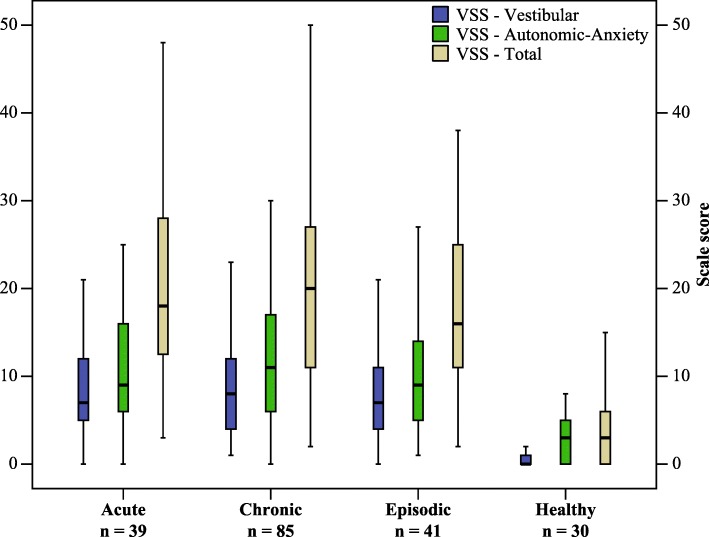
Shape and distribution of the scores in subgroups and healthy group. Note: Subgroups were classified based on the pattern of presentations of the vestibular symptoms at the time of rating. Abbreviation: VSS, Vertigo Symptom Scale

Table 4 shows the Spearman’s correlations between VSS **−** SF **− ** CK and its subscales, VAS **−** T, and TR **−** T (Pearson’s correlations revealed similar results). The Mann-Whitney U test compared the medians of the scores and revealed that the distributions were similar in all scales across subgroups (*ps* > .05). However, they were not similar when the mean ranks of the control group were compared to that of the subgroups and total patients (*ps* < .05). For Pearson’s correlations and the medians/interquartile ranges, see Additional file 4: Tables S3 and Table S4. Further, the shapes of the scores are shown in Fig. 3.

**Table 4:** Spearman’s correlation of the scales with the comparators

	n = 165		*n* = 159	*n* = 143
	VSS-V	VSS-AA	VAS-T	TR-T
VSS-V			0.48^a^	**− 0.37** ^**a**^
VSS-AA	0.58^a^		0.52^a^	**−0.14** ^**b**^
VSS-T	0.85^a^	0.91^a^	**0.57** ^**a**^	−0.27^a^

**Note:** Correlations stated in the hypotheses are in bold; ^a^Correlations is significant at the level of 0.01; ^b^Correlation is significant at the level 0.05

**Abbreviations:** *VSS–V/AA/T* Vertigo Symptom Scale–Vestibular/Autonomic-Anxiety/Total, *VAS–T* Visual Analogue Scale–Total, *TR–T* Tandem Romberg–Total

## Discussion

The study utilized a regulated process of cross-cultural adaptation and produced a VSS **−** SF **−** CK. The steps as described in the methodology were mostly applied in accordance with the related guidelines.

The nature of both the population and sample obliged the authors to involve raters (interviewers) and transform the instrument, as necessary, from self-administered to interviewer-administered (e.g., in cases of non-motivated and illiterate participants). The reliabilities of the VSS **−** SF − CK and the comparators were enhanced by these measures which was consistent with the test-retest results of the Norwegian and Japanese versions.

The results of both DWLS and PAF were nearly similar during EFA: seven items (1, 4, 6, 8, 10, 13, and 15), which are directly related to VD, firmly loaded onto vestibular factor with weak cross-loadings to the autonomic-anxiety factor; this was a preliminary sign of the discriminant ability of the VSS **−** V.

Previous studies as well as the present survey have used various types of analyses and samples; however, across these samples, two items (items-3 and 12) were associated with loading issues.

In five previous samples (Mexican, U.K. hospital, U.K. primary care, Norwegian [Table 3], and Japanese), item**−** 3 (nausea, vomiting) loaded interchangeably on both factors with noticeable cross-loadings on every occasion [15, 16, 46]. The mean loading (calculated by the authors) in these samples showed that the reflective**–**effect of anxiety factor on item**–**3 (loading 0.41) was higher than that of vestibular (loading 0.35).

The story of item**–**3 began when the original developer, for several reasons, intentionally decided to retain the item along with other items in the VSS **−** V [46], knowing that this item originally belonged to the VSS **−** AA from a physiological viewpoint [47]. However, the present sample has strongly placed the item into the VSS **−** AA (Table 3), which can be attributed to the heterogeneous nature of the symptoms in this sample; that is, various presentations and durations.

The item**−** 12 cross-loading issue (feeling faint, about to black out) is perhaps a structural matter. Out of six samples including the present survey, four of them included item**−** 12 correctly with VSS **−** AA [15, 16, 46]; the order, starting from weaker cross-loadings, was U.K. primary care, Japanese, U.K. hospital, and then the present sample. In the remaining two samples, the item unexpectedly settled on VSS **−** V; the order, starting from stronger loadings, was Norwegian then Mexican. It is unexpected for an item to oscillate or cross-load between constructs unless it is flawed. Accordingly, we believe this item represents two different types of symptoms. The words are clear and assumed to belong to the autonomic-anxiety symptoms; however, we noticed that some patients tried using many words or clauses to describe strange feelings of dizziness (spatial disorientation), words that were similar to those used to describe fainting and/or being about to black out. In spite of this, in the present study, item**−** 12 loaded adequately on VSS **−** AA (0.45); however, it was the only item characterized by the lowest loading and the highest cross-loading. The situation was investigated by deleting item**−** 12, which resulted (in both methods) in deflation of IFC and slight inflation of AVE by VSS **−** AA (Table 3). Consequently, the Fornell-Larcker criterion was also obtained for VSS **−** AA, leading to establishment of discriminant validity.

Regarding the 15 items’ structural consistency, the item loading results in both methods were nearly similar, but the robustness of polychoric correlation via DWLS was evident through higher AVE and item-loadings. The two-factor model in the VSS **−** SF **− **CK was suitable according to the recommended fit indices. Along with structure, the construct was also validated across internal consistency parameters such as αs, rhoA, and rhoC, and it was clear from the results that all values achieved desirable levels. Despite the low AVE, discriminant validity was also established by both HTMT and HTMT-inference, while the Fornell-Larcker criterion was obtained for only one factor, VSS **−** V.

The hypotheses regarding convergent validity were supported. An adequate positive correlation was found between VSS − T and VAS − T as well as a moderate negative correlation between the VSS **−** V and stability; the latter replicated a similar correlation (between VSS-V and path length) in a previous analysis [15]. Although the types of scores in VSS − AA and TR − T are different (subjective and objective), the resultant weak negative correlation between them (− 0.14) indicates the divergent ability of the VSS **−** AA because they measure two different constructs (anxiety and stability).

The instrument significantly discriminated the healthy group from the patients’ group and subgroups; however, it was not efficient in discriminating presentation subgroups, most probably because patients narrated the sum of their symptoms from the onset, regardless of the presence or absence of symptoms at the time of rating; as Yardley stated, the score is a cumulative measure [10]. The interpretability and responsiveness were beyond the scope of this study.

### Strengths and limitations

We believe that the study’s strength is its sample being representative of the target population. However, a potential limitation was related to convergent validity, as there were no validated comparator PROMs in Kurdish that could measure the same construct; for that reason we utilized VAS and emphasized discriminant validity. Second, close observation was required to sustain patients’ motivation for self-rating; and finally, because of the accommodation issue, we were obliged to shorten the minimum interval between rating events to 1 day.

## Conclusion

The VSS − SF was cross-culturally adapted to Kurdish. It revealed high external reliabilities. The structure of the 2-factor model was associated with high internal consistency and composite reliability with the ability to discriminate two latent variables (vestibular and autonomic-anxiety). These stabilities were confirmed by goodness of fit indices. It has adequate correlations with the comparators, demonstrating convergent validity. VSS − SF − CK is, then, a consistent and validated PROM that can be used by Kurdish researchers and clinicians to quantify vestibular symptoms before and/or after treatment protocols.

## Additional files

Additional file 1: Methodology and statistics. More detail on: Kurdish population, Cross-cultural adaptation, comparator instruments, external reliability test, and statistical approach. (DOCX 29 kb)

Additional file 2: Subjective ratings of contents and cultural understandability . A specific form designed for subjective rating for the consistency of the contents of each of the 15 translated symptoms in regard to meaning, lucidity, and cultural understandability. (PDF 1015 kb)

Additional file 3: VSS − SF − CK. The final version of the Vertigo Symptom Scale–Short form in central Kurdish dialect. (PDF 803 kb)

Additional file 4: **Table S1.** Horn’s Parallel analysis. **Table S2.** Minimum average partial. **Table S3.** Pearson’s correlations. **Table S4.** Medians and interquartile range of the scales. (DOCX 25 kb)

Additional file 5: Estimation of the AVE and rhoC. contains equation to estimate the average variance extracted (AVE) and composite reliability (rhoC), with the URL of the explaining movie. (XLSX 13 kb)

Additional file 6: Exploratory factor analysis by DWLS, Output of the FACTOR: A computer program, containing the detail of the exploratory factor analysis using polychoric correlation through robust diagonally weighted least squares. (TXT 29 kb)

Additional file 7: Codes and abbreviations of dataset. Description: Clarification of the codes and abbreviations of the dataset supporting the conclusion. (XLSX 11 kb)

Additional file 8: Dataset. Dataset supporting the conclusions of this article (XLSX 37 kb)

## Data Availability

The datasets supporting the conclusions of this article are included within the article and its Additional files 7 and 8.

## Abbreviations

AIC: Average Inter-item Correlation
AIID: Alpha If Item Deleted
AVE: Average Variance Extracted
CFI: Comparative Fit Index
CI-TC: Corrected Item-Total Correlation
DWLS: Diagonally Weighted Least Squares
EFA: Exploratory Factor Analysis
HPA: Horn’s Parallel Analysis
HTMT: Heterotrait–monotrait ratio
ICC: Intraclass Correlation Coefficient
IFC/IFC2: Inter-Factor Correlation/Square of IFC
MAP: Minimum Average Partial
PAF: Principal Axis Factoring
PC: Polychoric Correlations
PROMs: Patient-Reported Outcome Measures
rhoA: Reliability measure of the partial least squares
rhoC: Composite reliability
RMSEA: Root Mean Square Error of Approximation
TR-T: Tandem Romberg-Total
VAS-T: Visual Analogue Scale-Total
VD: Vestibular Disorders
VS: Vestibular Symptoms
VSS-SF-CK/V/AA/T: Vertigo Symptom Scale–Short Form-Central Kurdish/vestibular/Autonomic-Anxiety/Total
α: Cronbach’s alpha
