# Correction to: Cross-cultural adaptation, reliability, and validity of the Vertigo symptom scale–short form in the central Kurdish dialect

**DOI:** 10.1186/s12955-019-1209-7

**Published:** 2019-08-09

**Authors:** Sherko Saeed F. Zmnako, Yousif Ibrahim Chalabi

**Affiliations:** Department of Surgery-Otolaryngology, College of Medicine, University of Sulaimani, Presidency 1 Tasluja Street 501, P.O. Box: 334, Sulaimani, Kurdistan Region Iraq

**Correction to: Health Qual Life Outcomes (2019) 17:125**

**https://doi.org/10.1186/s12955-019-1168-z**

The original article [1] contained a minor typo in Fig. 1 affecting the motif ‘VSS-SF-CK’; this has since been corrected.
