# Supplementary material for: Cross-cultural adaptation, reliability, and validity of the Vertigo symptom scale–short form in the central Kurdish dialect
Source: Health Qual Life Outcomes. 2019 Jul 17;17:125. doi: 10.1186/s12955-019-1168-z (PMC6637568; doi:10.1186/s12955-019-1168-z)
Supplement: Supplementary file 1 — Methodology and statistics. More detail on: Kurdish population, Cross-cultural adaptation, comparator instruments, external reliability test, and statistical approach. (DOCX 29 kb) [file 12955_2019_1168_MOESM1_ESM.docx]

**Additional file 1**

**Methods and statistics**

***Population and Preparations***

*Population:* Kurds populate a wide area in the Middle East. There is a wide discrepancy in estimates of the total number of Kurds, which range broadly between 15 to 25 million. Kurdish is a member of the Indo-European family of languages, and is now official in Iraq; it consists of two main dialects: Kurdish Central (Sorani) and Kurdish Northern (Kurmanji) [1].

## Cross-cultural adaptation (CCA)

*Reconciliation:* to create a pre-final copy, in two consecutive sessions, the focus group (FG), in the presence of the first translator, compared and resolved differences between T1 and T2. Controversies were resolved by majority opinion.

*Back translation:* To examine the quality, the T12 was back-translated to the original language by a different licensed translator. During the review, the FG resolved four noticed discrepancies:

1. The Kurdish word used for “very often” was not explicit and was enforced by a popular word of Arabic origin.
2. To explain the word “spell,” two Kurdish words were used.
3. A clause was added to clarify the meaning of “dizziness.”
4. A popular Arabic word was inserted in brackets to define the word “unsteady.”

***Comparator instruments:***

*Visual analogue scale (VAS):* This has good psychometric properties [2]. A percentage rating, in other words, choosing a specified number as a fraction of hundred is a widely adopted tool used by the majority of people in this locality, even those who are illiterate. Hence, a visual analogue scale was applied.

*Tandem Romberg (TR):* A printed figure of two straight feet one in front of the other (toe to heel) without angulation glued on a stable flat ground. The test was carried out in a noiseless room; so that, the patient unable to get benefit from auditory information to maintain balance in eyes closed conditions. Participants were asked to stand quietly on the figure, each palm over the opposite shoulder looking forward. Three trials were administered for each of the mentioned (in the main study) four conditions. Times for each trial were calculated from beginning to end using a stopwatch. The beginning was considered to be when the patient adopted the condition and s/he was ready. While, the end was identified as comprising the following five situations: 1- when the participant could complete 60 seconds successfully; or failed to complete when s/he: 2- moved palm or foot; 3- lost balance; 4- sought assistance (holding objects); 5- opened eyes in eyes closed conditions. Only one trial was administered for each condition if the patient could complete 60 seconds successfully. Moreover, the third trial was only administered when the patient could not complete the first and the second trials. Number of seconds in the administered trial or trials in each condition were summed out of 60 seconds, the total sum of all four conditions were summed out of 240 seconds [3].

***External reliability tests***

Two raters (R1 and R2) were involved. The reliability group of VSS-SF (n = 74) answered the 15 questions on two separate occasions (O1 and O2). The time interval between ratings was one to five days, the timing of O2 was arranged by the raters while the patient returned to receive their results from the investigations or to repeat their rehabilitation protocols. From these, 56 were randomly assigned for intra-rater tests (each subject was rated by the same rater on both occasions); 28 and 28 were rated by R1 and R2, respectively. The remaining 18 were enrolled for inter-rater tests (the subject was rated by both raters, each for one occasion) (table 2).

To avoid missing values during rating, systematic non-reply of one of the responses especially (never = 0) was prevented [4]. Test-retest reliability was examined by comparing the results of both occasions. The comparator instruments were also exposed to the recommended regulations. However, the time interval between the two occasions for Tandem Romberg was 1 to 2 hours to remove the effect of in-between rehabilitation.

***Statistics***

*External reliability:* The selection of raters (fixed) in this study governed intraclass correlation coefficient (ICC). The two-way mixed-effect (model), mean of k raters (type), and absolute agreement (definition) were used to evaluate all types of reliability tests[5].

*Construct validity:* Because there is no gold standard in the field of vestibular disorders [6], the authors validated the construct instead of the criterion.

The partial least squares path modeling (PLS) is a stable statistic. Although it is a variance-based structural modelling, it can keep Type I error down in a non-normal distribution [7, 8]. SmartPLS software provides sufficient results in respect of construct and discriminant validity [9]. That is, PLS is also involved in CFA; yet, to agree with the purpose of the current study, the reflective measurement model (causality), default setting, and PLS algorithm were set.

*Discriminating (discriminative) validity*: Due to the ordinal nature of the data and non-normality, to determine this validity, methodologists recommend using medians instead of means and standard deviations [10]; hence, it was determined by Mann-Whitney test which compared the medians of the scores in the three subgroups because the shapes of the their scales were similar. However, mean ranks were compared through the default Mann-Whitney test when control group was compared with the subgroups and the total patients because the shapes of their scales were not similar [11].

**References**

1. Haig G, Matras Y. Kurdish linguistics: a brief overview. STUF-Language Typology and Universals. 2002;55(1):3–14.

2. de Boer AG, van Lanschot JJ, Stalmeier PF, van Sandick JW, Hulscher JB, de Haes JC, et al. Is a single-item visual analogue scale as valid, reliable and responsive as multi-item scales in measuring quality of life? Qual Life Res. 2004;13(2):311–20.

3. Johnson BG, Wright AD, Beazley MF, Harvey TC, Hillenbrand P, Imray CH, et al. The sharpened Romberg test for assessing ataxia in mild acute mountain sickness. Wilderness Environ Med. 2005;16(2):62-6.

4. Holmberg J, Karlberg M, Harlacher U, Rivano-Fischer M, Magnusson M. Treatment of phobic postural vertigo. A controlled study of cognitive-behavioral therapy and self-controlled desensitization. J Neurol. 2006;253(4):500–6.

5. Koo TK, Li MY. A Guideline of selecting and reporting intraclass correlation coefficients for reliability research. J Chiropr Med. 2016;15(2):155–63.

6. Furman JM. Role of posturography in the management of vestibular patients. Otolaryngol Head Neck Surg. 1995;112(1):8–15.

7. Dijkstra TK, Henseler J. Consistent Partial Least Squares Path Modeling. MIS quarterly. 2015;39(2).

8. Henseler J. Partial least squares path modeling. In: Leeflang PSH, Wieringa JE, Bijmolt THA, Pauwels KH, editors. Advanced methods for modeling markets. Basel: Springer International Publishing; 2017. p. 361–81.

9. Ringle CM, Wende S, Becker J-M. SmartPLS3. Bönningstedt, <http://www.smartpls.com> 2015.

10. de Vet HCW, Terwee CB, Mokkink LB, Knol DL. Validity. In: de Vet HCW, Terwee CB, Mokkink LB, Knol DL, editors. Measurement in medicine: a practical Guide. New York: Cambridge University Press; 2011. p. 150–201.

11. Marshall E, Boggis E. The Statistics Tutor’s Quick Guide to Commonly Used Statistical Tests, <http://www.statstutor.ac.uk/resources/uploaded/tutorsquickguidetostatistics.pdf> (2016).
