# Supplementary material for: Cross-cultural adaptation, reliability, and validity of the Vertigo symptom scale–short form in the central Kurdish dialect
Source: Health Qual Life Outcomes. 2019 Jul 17;17:125. doi: 10.1186/s12955-019-1168-z (PMC6637568; doi:10.1186/s12955-019-1168-z)
Supplement: Supplementary file 2 — Subjective ratings of contents and cultural understandability . A specific form designed for subjective rating for the consistency of the contents of each of the 15 translated symptoms in regard to meaning, lucidity, and cultural understandability. (PDF 1015 kb) [file 12955_2019_1168_MOESM2_ESM.pdf]

دیاریکردنی وەلام بۆ چەندجارە توشبونی ئەم (١٥) سکاڵاینەی خوارەوە و ئەژمارکردنی بەهای ئەو وەڵامانە بە کاردەهێنرێ وەک پێوەرێک بۆ دیاریکردنی ڕاددەی هێزی سکاڵاکانی سەرەسورە، بۆ هەر پرسیارێک تەنها پێنج وەڵام هەیە، و هەر وەڵامێکیش بەهایەکی بۆدانراوە بەم شێوەیە؛(هیچ کات=٠، کەمجار=١ ، هەندێجار=٢، زۆرجار=٣، هەموکات=٤). نەخۆش دەبێت نەنها یەک وەڵام بۆ هەر پرسیارێک هەڵبژێرێت.

هەریەک لەم ١٥ سکالایەش پێوەرێک بەرامبەری هەیە، تکایە لە ڕوانگەی کلتوریی زمانی کوردی ناوەندەوە (سۆرانی)، بە دیاری کردنی نیسبەتی سەددی لەسەر پێوەرەکان ڕاو و بۆچونی خۆت دیاری بکە لە سەر شێوازی داڕشتن و وشەکانی هەر سکالایەک، لە بوارەکانی: توندو تۆڵی ,ڕۆشنی, زمانەوانی و هەروەها تێگەشتنی زۆربەی خەڵک لە مەبەستی سکالاکان و وەڵامەکانیان کە لە سەرەوە ئاماژەی بۆکرا . کەموکوڕیت بەدیکرد، تکایە دیاری بکە و شێوازی گونجاو ترمان بۆ بنوسە. لە بەشی سەرەوەی یەکەم پێوەر چەند مەودایەک بۆ وەڵامدانەوە دیاری کراوە بە نیسبەتی سەددی، تکایە سودمەند بە لەو مەودایانە کاتێک نیسبەکە دیاری دەکەیت.

Answers of the following (15) symptoms are used as a score to measure the level of impact that produced by vestibular disorders, for each symptom there are five established answers, and each answer has a specific value; ;(never=0, a few times=2 several times=3, quite often=3 and very often=4). Patient must select only one answer for each symptom.

Please identify on each scale, your subjective percentage rating for the consistency of the contents of each of the following 15 translated symptoms in regard of meaning, lucidity and cultural understandability. Refer to the identified range of response located above the scales.
Notes: In addition to rating, patients must answer each item by selecting one of the aforementioned values; Members of the focus group must compare translated symptoms with the original one.

<table>
<tr><th colspan="2">سکاڵاکان</th><th>باش نی یە
کەمتر لە ٥٠%</th><th>مامناوەند
لە نێوان ٥٠ – ٧٥%</th><th>باش
لە نێوان ٧٥- ٩٠%</th><th>نایاب
زیاتر لە ٩٠%</th></tr>
<tr><th colspan="2">Symptoms</th><th>Poor
Less than 50%</th><th>Moderate
between
50-75%</th><th>Good
Between
75-90%</th><th>Excellent
more than
90%</th></tr>
<tr><td>1</td><td>هەستکردن کە خۆت یان شتەکانی دەوروبەرت دەسوڕێنەوە یان دەجوڵێن بۆ ماوەی کەمتر لە (٢٠) دەقەە
A feeling that either you, or things around you, are spinning or moving, lasting less than 20 minutes</td><td colspan="4">0% 5 10% 15 20% 25 30% 35 40% 45 50% 55 60% 65 70% 75 80% 85 90% 95 100%</td></tr>
<tr><td>2</td><td>نۆبەی گەرما یان سەرما
Hot or cold spells</td><td colspan="4">0% 5 10% 15 20% 25 30% 35 40% 45 50% 55 60% 65 70% 75 80% 85 90% 95 100%</td></tr>
<tr><td>3</td><td>دڵ تێکهەڵاتن ، ڕشانەوە
Nausea (feeling sick), vomiting</td><td colspan="4">0% 5 10% 15 20% 25 30% 35 40% 45 50% 55 60% 65 70% 75 80% 85 90% 95 100%</td></tr>
<tr><td>4</td><td>هەستکردن کە خۆت یان شتەکانی دەوروبەرت دەسوڕێنەوە یان دەجوڵێن بۆ ماوەی زیاتر لە (٢٠) دەقەە
A feeling that either you, or things around you, are spinning or moving, lasting more than 20 minutes</td><td colspan="4">0% 5 10% 15 20% 25 30% 35 40% 45 50% 55 60% 65 70% 75 80% 85 90% 95 100%</td></tr>
<tr><td>5</td><td>دڵ پەلەپەل کردن یان دڵەکوتێ
Heart pounding or fluttering</td><td colspan="4">0% 5 10% 15 20% 25 30% 35 40% 45 50% 55 60% 65 70% 75 80% 85 90% 95 100%</td></tr>
<tr><td>6</td><td>هەستکردن بەوەی کە گێژی یان وڕی یان بە سەر عەرزەوە نیت بە درێژایی ڕۆژ
A feeling of being dizzy, disoriented or “swimmy” lasting all day</td><td colspan="4">0% 5 10% 15 20% 25 30% 35 40% 45 50% 55 60% 65 70% 75 80% 85 90% 95 100%</td></tr>
<tr><td>7</td><td>سەرئێشە یان هەستکردن بەوەی سەرت قورسە
Headache, or feeling of pressure in the head</td><td colspan="4">0% 5 10% 15 20% 25 30% 35 40% 45 50% 55 60% 65 70% 75 80% 85 90% 95 100%</td></tr>
<tr><td>8</td><td>نەتوانی بەباشی بەپێوە بوەستیت یان ڕێ بکەیت بە بێ دەستگرتن یان یارمەتی، ڕەتڵدان یان بەلاداکەوتن</td><td colspan="4">0% 5 10% 15 20% 25 30% 35 40% 45 50% 55 60% 65 70% 75 80% 85 90% 95 100%</td></tr>
</table>

	Unable to stand or walk properly without support, veering or staggering to one side	
9	هەناسەتوندی یان هەناسەسواری<br>Difficulty breathing, been short of breath	0% 5 10% 15 20% 25 30% 35 40% 45 50% 55 60% 65 70% 75 80% 85 90% 95 100%
10	هەستکردن بە ناجێگیری، خەریک بێت تەوازن لە دەست بدەیت بۆ ماوەی زیاتر لە (٢٠) دەققە<br>Feeling unsteady, about to lose balance, lasting more than 20 minutes	0% 5 10% 15 20% 25 30% 35 40% 45 50% 55 60% 65 70% 75 80% 85 90% 95 100%
11	ئارەقەکردنەوەی زۆر<br>Excessive sweating	0% 5 10% 15 20% 25 30% 35 40% 45 50% 55 60% 65 70% 75 80% 85 90% 95 100%
12	هەستکردن بە بێ هێزی ، خەریک بێت ببورێیتەوە<br>Feeling faint, about to black out	0% 5 10% 15 20% 25 30% 35 40% 45 50% 55 60% 65 70% 75 80% 85 90% 95 100%
13	هەستکردن بە ناجێگیری، خەریک بێت تەوازن لە دەست بدەیت بۆ ماوەی کەمتر لە (٢٠) دەققە<br>Feeling unsteady, about to lose balance, lasting less than 20 minutes	0% 5 10% 15 20% 25 30% 35 40% 45 50% 55 60% 65 70% 75 80% 85 90% 95 100%
14	ئازاری دڵ یان سنگ<br>Pains in the heart or chest region	0% 5 10% 15 20% 25 30% 35 40% 45 50% 55 60% 65 70% 75 80% 85 90% 95 100%
15	هەستکردن بەوەی کە گێژی یان وڕی یان بە سەر عەرزەوە نیت بۆ ماوەی کەمتر لە (٢٠) دەققە<br>A feeling of being dizzy disoriented or "swimmy", lasting less than 20 minutes	0% 5 10% 15 20% 25 30% 35 40% 45 50% 55 60% 65 70% 75 80% 85 90% 95 100%
