# Supplementary material for: Cross-cultural adaptation, reliability, and validity of the Vertigo symptom scale–short form in the central Kurdish dialect
Source: Health Qual Life Outcomes. 2019 Jul 17;17:125. doi: 10.1186/s12955-019-1168-z (PMC6637568; doi:10.1186/s12955-019-1168-z)
Supplement: Supplementary file 3 — VSS − SF − CK. The final version of the Vertigo Symptom Scale–Short form in central Kurdish dialect. (PDF 803 kb) [file 12955_2019_1168_MOESM3_ESM.pdf]

ناوی نەخۆش:	ڕەگەز:	تەمەن:
بەروار:	ID:	

Vertigo Symptom Scale-Short form-Kurdish-central (VSS-SF-KC)
پێوەری سکاڵای سەرەخولێ- فۆرمی کورت

حەز دەکەین بزانین، کامانەن ئەو سکاڵایانەی کە پەیوەندیان بە گێژبونەوە هەبووە و ئێوە هەتانبووە لەم ماوەیەی پێشودا، تکایە ژمارەی گونجاو دیاری بکە بەرامبەر هەر سکاڵایەک لەم لیستەی خوارەوەدا، بۆ ئەوەی دەربکەوێت کە چەندجار هەتانبوە لە ماوەی مانگی پێشودا، توندی هەر ژمارەیەک لەخوارەوە دیاری کراوە

٠ یەعنی هیچ کات | ١ یەعنی کەمجار | ٢ یەعنی هەندێ جار | ٣ یەعنی زۆرجار | ٤ یەعنی هەموکات (دایم)

	سکاڵا	هیچ کات	کەمجار	هەندێ جار	زۆرجار	دایم
١	هەستکردن کە خۆت یان شتەکانی دەوروبەرت دەسوڕێنەوە یان دەجولێن بۆ ماوەی کەمتر لە (٢٠) دەقە	٠	١	٢	٣	٤
٢	نۆبەی گەرما یان سەرما (تاوی گەرما یان سەرما)	٠	١	٢	٣	٤
٣	دڵ تێکهەڵاتن ، ڕشانەوە	٠	١	٢	٣	٤
٤	هەستکردن کە خۆت یان شتەکانی دەوروبەرت دەسوڕێنەوە یان دەجولێن بۆ ماوەی زیاتر لە (٢٠) دەقە	٠	١	٢	٣	٤
٥	دڵ پەلەپەل کردن یان دڵەکوتێ	٠	١	٢	٣	٤
٦	هەستکردن بەوەی کە گێژی یان وڕی (بە سەر عەرزەوە نیت) بە درێژایی ڕۆژ	٠	١	٢	٣	٤
٧	سەرئێشە یان هەستکردن بەوەی سەرت قورسە	٠	١	٢	٣	٤
٨	نەتوانی بەباشی بەپێوە بوەستیت یان ڕێ بکەیت بە بێ دەست گرتن یان یارمەتی، ڕەتڵدان یان بەلاداکەوتن	٠	١	٢	٣	٤
٩	هەناسەتوندی یان هەناسەسواری	٠	١	٢	٣	٤
١٠	هەستکردن بە ناجێگیری، خەریک بێت هاوسەنگی (تەوازن) لە دەست بدەیت بۆ ماوەی زیاتر لە (٢٠) دەقە	٠	١	٢	٣	٤
١١	ئارەقەکردنەوەی زۆر	٠	١	٢	٣	٤
١٢	هەستکردن بە بێ هێزی ، خەریک بێت ببورێیتەوە	٠	١	٢	٣	٤
١٣	هەستکردن بە ناجێگیری، خەریک بێت هاوسەنگی (تەوازن) لە دەست بدەیت بۆ ماوەی کەمتر لە (٢٠) دەقە	٠	١	٢	٣	٤
١٤	ئازاری دڵ یان سنگ	٠	١	٢	٣	٤
١٥	هەستکردن بەوەی کە گێژی یان وڕی (بە سەر عەرزەوە نیت) بۆ ماوەی کەمتر لە (٢٠) دەقە	٠	١	٢	٣	٤
