# Supplementary material for: Cross-cultural adaptation, reliability, and validity of the Vertigo symptom scale–short form in the central Kurdish dialect
Source: Health Qual Life Outcomes. 2019 Jul 17;17:125. doi: 10.1186/s12955-019-1168-z (PMC6637568; doi:10.1186/s12955-019-1168-z)
Supplement: Supplementary file 4 — Table S1. Horn’s Parallel analysis. Table S2. Minimum average partial. Table S3. Pearson’s correlations. Table S4. Medians and interquartile range of the scales. (DOCX 25 kb) [file 12955_2019_1168_MOESM4_ESM.docx]

**Additional file 4**

***Horn’s Parallel analysis (HPA) and Minimum average partial (MAP)***

To implement factor solution, the ideal number of the components should be identified. Guidelines recommend various strategies in this regard [1, 2] such as: scree plot, a priori hypothesis, HPA [3], and MAP [4]. The following results were generated from SPSS syntax for HPA and MAP, which suggested 2−factors solution (values were presented as generated from the syntaxes).

**Table S1.** Generated data from the syntax of parallel analysis

| **Component** | **Raw data Eigenvalue** | **Mean** | **Random data Eigenvalue** |
| --- | --- | --- | --- |
| 1 | 4.728047 | .646551 | .793333 |
| 2 | .914256 | .513729 | .611101 |
| 3 | .463210 | .413163 | .492010 |
| 4 | 342032 | .321345 | .387580 |
| 5 | 254970 | 251837 | 326927 |
| 6 | .126855 | .176483 | .231430 |
| 7 | .087036 | .113714 | .168414 |
| 8 | -.026161 | .052747 | .108982 |
| 9 | -.052582 | -.008164 | .037287 |
| 10 | -.104527 | -.064487 | -.020644 |
| 11 | -.136085 | -119741 | -.071196 |
| 12 | -.164388 | -.176355 | -.144788 |
| 13 | -.176476 | -.228860 | -.196435 |
| 14 | -.219167 | -.281834 | -.249937 |
| 15 | -.282802 | -.342770 | -.298055 |

**Note:** Raw data permutation in principal axis factoring showed that the Eigenvalues of the raw data is greater than that of the percentile random data only in the first and second components; that is, the suggested number of components is: 2.

**Table S2.** Generated data from the syntax of minimum average partial

| **Eigenvalues** | **Component** | **Squared** | **Fourth power** |
| --- | --- | --- | --- |
| 4.6729 | .0000 | .3125 | .1551 |
| 1.7710 | 1.0000 | .2451 | .0736 |
| .4810 | 2.0000 | .0664 | .0119 |
| .4214 | 3.0000 | .1276 | .0519 |
| .2332 | 4.0000 | .2042 | .1160 |
| .1867 | 5.0000 | .2718 | .1526 |
| .1373 | 6.0000 | .4346 | .3312 |
| .0965 | 7.0000 | 1.0000 | 1.0000 |
| **Note:** Velicer's minimum average partial test; The smallest average squared partial correlation is: 0.0664; The smallest average fourth power partial correlation is: 0.0119; The number of components according to the original (1976) MAP test is: 2; The number of components according to the revised (2000) MAP test is: 2 (these notes were generated from the syntax). | | | |

***Pearson's correlation***

**Table S3.** Pearson's correlation of the scales with the comparators

|  | n = 165 | | n = 159 | n = 143 |
| --- | --- | --- | --- | --- |
|  | **VSS-V** | **VSS-AA** | **VAS-T** | **TR-T** |
| VSS-V |  |  | 0.47^a^ | **-0.42^a^** |
| VSS-AA | 0.58^a^ |  | 0.50^a^ | -0.17^b^ |
| VSS-T | 0.87^a^ | 0.91^a^ | **0.55^a^** | -0.32^a^ |

**Note:** Correlations stated in the hypotheses are in bold; ^a^Correlation is significant at the level of 0.01; ^b^Correlation is significant at the level 0.05.

**Abbreviations:** VSS-V/AA/T, Vertigo Symptom Scale-Short Form-Vestibular/Autonomic-Anxiety/Total; VAS-T, Visual Analogue Scale-Total; TR-T, Tandem Romberg-Total.

***Median and interquartile range of the scales***

**Table S4.** Median and interquartile range of the scales

|  | **Total patients** | | **Reliability subgroup** | | **Presentation subgroups^a^** | | | | | | **Healthy group** | |
| --- | --- | --- | --- | --- | --- | --- | --- | --- | --- | --- | --- | --- |
|  |  |  |  |  | **Acute** | | **chronic** | | **Episodic** | |  |  |
|  | n = 165 | | n = 76 | | n = 39 | | n = 85 | | n = 41 | | n = 30 | |
|  | M | IQR | M | IQR | M | IQR | M | IQR | M | IQR | M | IQR |
| VSS-V | 8 | 7 | 8 | 7 | 7 | 7 | 8 | 8 | 7 | 7 | **0** | **1** |
| VSS-AA | 10 | 10 | 10.5 | 11 | 9 | 10 | 11 | 11 | 9 | 10 | **3** | **5** |
| VSS-T | 18 | 16 | 18.5 | 18 | 18 | 16 | 20 | 17 | 16 | 15 | **3** | **6** |

**Note:** ^a^Nature of the symptoms at the time of rating, not related to disorders or syndromes; Bold values are median and IRQ of the healthy group.

**Abbreviations:** M, Median; IQR, Interquartile range; VSS-V/AA/T, Vertigo Symptom scale-short form-Vestibular/Autonomic-Anxiety/Total.

**References**

1. Watkins MW. Exploratory factor analysis: a guide to best practice. J Black Psychol. 2018;44(3):219–46.

2. Zygmont C, Smith MR. Robust factor analysis in the presence of normality violations, missing data, and outliers: Empirical questions and possible solutions. Tutor Quant Methods Psychol. 2014;10(1):40–55.

3. Garrido LE, Abad FJ, Ponsoda V. A new look at Horn's parallel analysis with ordinal variables. Psychol Methods. 2013;18(4):454–74.

4. O’connor BP. SPSS and SAS programs for determining the number of components using parallel analysis and Velicer’s MAP test. Behav Res Methods Instrum Comput. 2000;32(3):396–402.
